# Prevalence of human papillomavirus and its prognostic value in vulvar cancer: A systematic review and meta-analysis

**DOI:** 10.1371/journal.pone.0204162

**Published:** 2018-09-26

**Authors:** Jianxin Zhang, Yang Zhang, Zhenyu Zhang

**Affiliations:** Department of Obstetrics and Gynecology, Capital Medical University affiliated Beijing Chaoyang Hospital, Chaoyang District, Beijing, PR. China; Istituto Nazionale Tumori IRCCS Fondazione Pascale, ITALY

## Abstract

The purpose of this study was to estimate the prevalence of human papillomavirus (HPV) in vulvar cancer and determine whether positive HPV in vulvar cancer was associated with a better prognosis. Literature searches of Ovid EMBASE, PubMed, Web of Science and Cochrane Library were performed to identify related studies published from January 2000 to May 2017. A total of 33 studies including 7,721 subjects were selected in this meta-analysis. Overall, the HPV prevalence in vulvar cancer tissue was 34% (95% CI: 28%-39%) with 45% (95% CI: 28%-64%) in Asian populations and 34% (95% CI: 26%-42%) in Caucasian populations. The HPV-positive vulvar cancer was associated with better overall survival (hazard ratio = 0.64, 95% CI: 0.47–0.87; *P* = 0.004) and recurrence-free survival (hazard ratio = 0.66, 95% CI: 0.45–0.97; *P* = 0.03) compared with HPV-negative counterpart. HPV status may play an important role in predicting the prognosis of patients with vulvar cancer. The HPV-positive vulvar cancer women might relatively have a better survival than HPV-negative ones.

## Introduction

Vulvar cancer is the fourth most common type of gynecological cancer and encompasses approximately 6% of all female genital tract malignancies. According to cancer statistics, there have been more than 6,000 cases and 1,150 deaths every year in the United States [1]. Although the incidence of vulvar cancer is low, it has increased over the past few decades, particularly amongst younger women [2–6]. Although patients with early stage vulvar cancer have a favorable prognosis, the patients with advanced disease have poor treatment outcomes [7]. Therefore, it is important to improve the prognosis of vulvar cancer, especially for patients with advanced stage of vulvar cancer.

Ninety percent of vulvar cancers are squamous cell carcinomas [8, 9] and several other morphological variants mainly include basaloid, keratinising, warty and verrucous carcinoma [10]. One third of vulvar cancer cases are basaloid and warty variants, which are more common in younger women and are often associated with human papillomavirus (HPV) infection. On the contrary, keratinising variants caused by chronic vulvar dermatosis are not associated with HPV and predominantly occur in older women [11].

There are more than 100 types of HPV, and these are divided into 3 broad categories according to their oncogenic potential [12–14]. The association between HPV infection and some types of gynecological tumors have been identified in cervical, endometrial, and ovarian cancers. However, due to the low frequency of vulvar cancer, only a few detailed studies were focused on the effect of HPV infection on the survival outcomes in vulvar cancer patients. Therefore, the prognostic significance of HPV infection in vulvar cancer has not been fully understood yet and is still debatable. Some studies reported that patients with HPV-positive tumors have a better prognosis than those with HPV-negative tumors, whereas others did not reach the same conclusion. The HPV prevalence in vulvar cancer cases ranges in different studies from 3.3% to 76.5%; the inconsistent results of the prognostic value of HPV in vulvar cancer may be due to the diverging prevalence rates of HPV.

To our knowledge, the prevalence and prognostic value of HPV infection in vulvar cancer reported in previous studies have not been subjected to statistical pooling. In order to validate these personal observations, the aims of this study were to evaluate the HPV prevalence, determine the prognostic value of HPV and clarify which variables may be the underlying causes of heterogeneity in prevalence and prognostic value of HPV in vulvar cancer.

## Methods

### Data sources and search strategy

Literature searches of Ovid EMBASE databases, PubMed, Web of Science, and Cochrane Library databases were performed from January 2000 to May 5, 2017. The main keywords used for the search were “carcinoma or cancer or malignancy or adenocarcinoma or neoplasm or carcinoma” and “vulva or vulvar or genito-urinary or genitourinary or genital or genitalia or perineum or perineal” and “papilloma virus or papillomavirus or papillomavirus infections or HPV”. The detailed search terms and strategies are shown in S1 Table. The articles published were limited to English language. Additionally, the citation lists of retrieved articles were manually screened independently by two authors. All selected studies were checked according to a Newcastle-Ottawa Quality assessment Scale developed previously [15].

### Selection criteria

Inclusion criteria of this meta-analysis were as follows: (1) independently published study, investigating the prevalence of HPV in vulvar cancer patients; (2) a study investigated the association between HPV status and survival outcomes in vulvar cancer patients. The following exclusion criteria were also applied: (1) reviews; (2) case report or case series; (3) studies lacking enough information.

### Data extraction and quality assessment

Two investigators (Jianxin Zhang and Yang Zhang) independently performed the data extraction and quality assessment. The detailed information collected for each study mainly included first author, publication year, ethnicity, country, study period, types of vulvar cancer, type of tissue, HPV prevalence, HPV Types and detection methods, survival indicators, HR estimates, and follow-up time., The studies were merged into a unique extraction, if several publications were overlapped. If a study hadn`t reported the HR and its related 95%CI, Kaplan-Meier survival curves could be used referring to previously published methods [16, 17]. Additionally, the discrepancies were resolved via discussion.

The quality of each eligible study was assessed by the nine-star Newcastle–Ottawa Scale (NOS). A study would be considered to have high quality with the NOS score equal or greater than seven scores. After data extraction and assessment, the information would be examined and adjudicated independently by an investigator (Zhenyu Zhang).

### Statistical analysis

Of the studies identified, the overall prevalence of HPV in vulvar cancer was analyzed, using the R software (version 3.4.1) and was estimated based on a random-effects model, in which the between-study variance was determined with the Der-Simonian Laird estimator. The results of the overall prevalence of HPV in vulvar cancer for all studies sorted by first author were presented using forest plots. The prevalence of HPV among vulvar cancer patients from each study was presented with exact binomial 95% confidence intervals (CIs).

The meta-analysis of the prognostic value of HPV in vulvar cancer was carried out using Review Manager (RevMan) 5.3 analysis software (Copenhagen: The Nordic Cochrane Centre, The Cochrane Collaboration, 2014). The association between HPV and survival outcomes in patients with vulvar cancer was estimated by calculating pooled HRs and related 95% CI. If the eligible articles did not report the HRs and 95% CI, they would be extracted according to previously published methods. The results were presented using forest plots. The study heterogeneity was assessed and presented by *Chi*^*2*^ and *I*^*2*^. *I*^*2*^ values of 25, 50 and 75% were defined as low, moderate, and high estimates, respectively. If no study heterogeneity existed (*P*>0.10 or *I*^*2*^<50%), the meta-analysis would use the fixed-effects model; otherwise, use the random-effects model. A HR<1 indicated a better survival outcome for HPV-positive while HR>1 indicated a worse survival outcome for HPV-positive. A *P* value less than 0.05 was considered statistically different. The sensitivity analysis was performed by deleting each study in turn to assess the consistency and quality of the results. The funnel plot and Egger`s test were performed to assess the potential publication bias.

## Results

### Literature search and study selection

**Fig 1:**
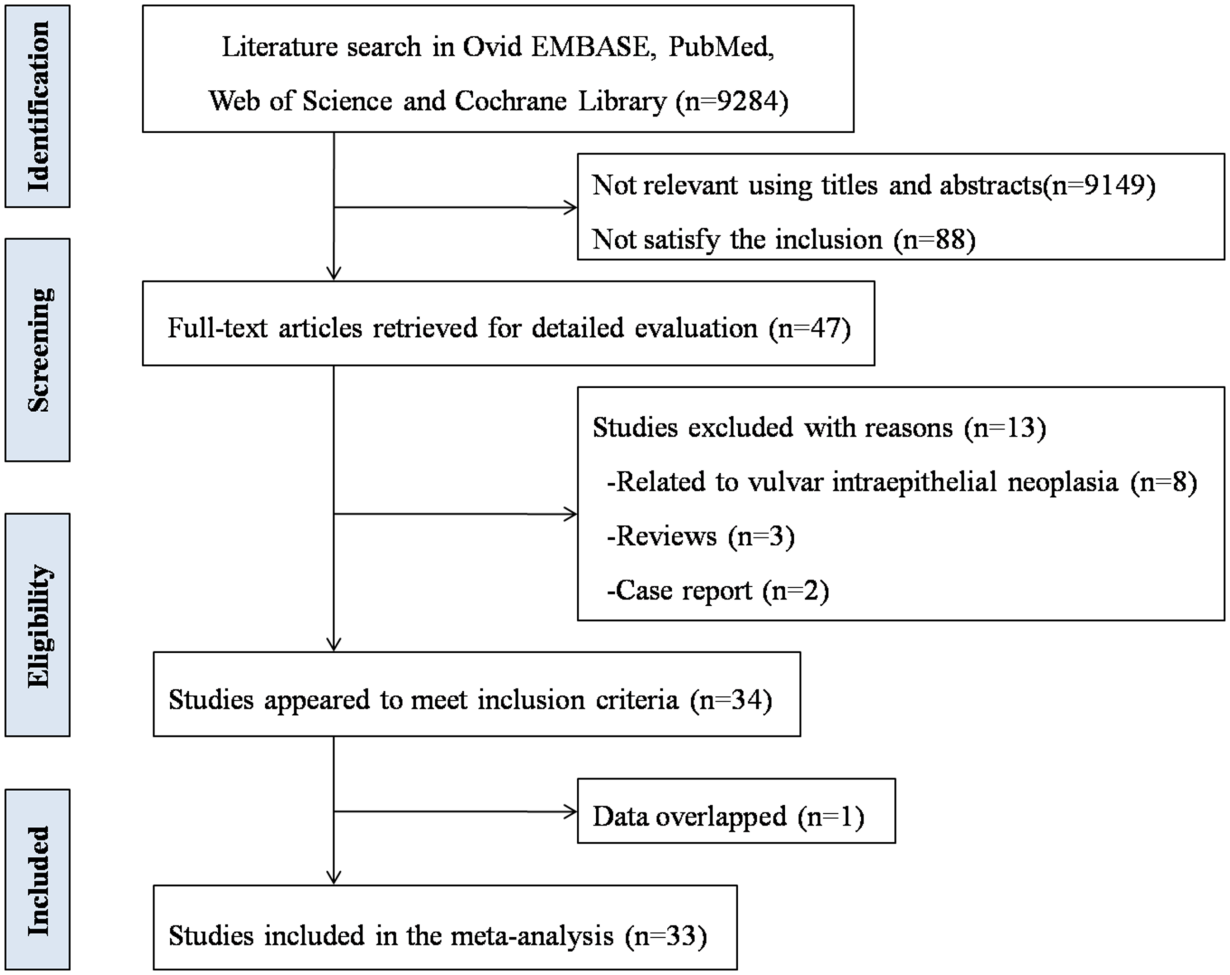
Flow chart of the selection process for the eligible studies.

The process of literature search and study selection is summarized in Fig 1. A total of 9,284 studies were collected using the detailed search strategies in the 4 databases selected. After reading the titles and abstracts, 47 potential studies were included for full-text view. With further screening, 33 studies, [18–50] reporting the HPV prevalence in vulvar cancer were identified according to the inclusion criteria. The main characteristics of the studies included are summarized in Table 1. There were 5 studies reported on Asian populations and 23 studies on Caucasian populations. The types of HPV in these studies mainly included HPV 6, 11, 16, 18, 31, 33. In addition, the majority of these eligible studies used the formalin-fixed paraffin-embedded tissue to perform the HPV detection and genotyping. The majority of these eligible studies used the PCR-based methods for the detection of HPV.

**Table 1:** The main characteristics of the 33 studies included in the meta-analysis.

Study	Ethnicity	Country	Study period	Study types	Cancer Types	Tissue Types	Quality assessment of samples	HPV Prevalence		HPV Types	Detection Methods
								n/N	%		
Alonso, et al (2011)	Caucasians	Spain	1995–2009	Hospital-based	VSCC	FFPE	β-globin PCR analysis	19/98	19.4%	16, 31, 33, 51, 52, 56	SPF-10 primers, INNO-LiPA HPV Genotyping kit
Antonets, et al (2013)	Caucasians	Russian	NA	NA	VC	FFPE	NA	12/58	20.6%	16, 18, 31, 33, 35, 45, 51, 52, 58, 59	PCR
Engelman, et al (2003)	Mixed	Brasil	1983–1995	Institution-based	IVSCC	FFPE	NA	4/55	7.3%	16/18	ISH
Felez-Sanchez, et al (2016)	Caucasians	Spain	NA	Institution-based	VSCC	FFPE	Tubulin PCR analysis	30/902	3.3%	2, 16, 33, 45, 52, 53, 54, 66, 70, 74	PCR-SPF10/DEIA/LiPA25
Fuste, et al (2010)	Caucasians	Spain	1990–2007	Institution-based	VSCC	NA	NA	18/94	19%	16	PCR
Gargano, et al (2012)	Caucasians	United States	1995–2005	Registry-based	IVC	FFPE	β-globin PCR analysis	121/176	68.8%	16,18,31,33,45,52,59	PCR-PGMY9/11 primers and type-specific hybridization; retesting with SPF10
Hampl, et al (2008)	Caucasians	Germany	1980–2007	Institution-based	VC	NA	NA	18/36	50%	6, 11, 16, 18, 33, 42, 52, 55	PCR
Huang, et al (2005)	Asians	China	NA	Institution-based	VSCC	Frozen	β-globin PCR analysis	6/8	75%	16, 18	PCR
Karnezis, et al (2015)	Caucasians	Canada	1985–2005	Hospital-based	VSCC	FFPE	NA	77/193	40%	NA	NA
Kim, et al (2015)	Asians	Korea	1998–2011	Institution-based	VC	FFPE/Frozen	NA	15/35	42.86%	16, 18, 31, 33, 35, 39, 45, 51, 52, 56, 58, 59, 68	HC2 test
Kowalewska, et al (2010)	Caucasians	Poland	2003–2006	Institution-based	VSCC	FFPE	β-globin PCR analysis	7/46	15%	6, 16, 58	Linear Array HPV Detection Kit
Koyamatsu, et al (2003)	Asians	Japan	1982–1998	Institution-based	VC	FFPE	NA	4/31	12.8%	16,18	PCR
Larsson, et al (2012)	Caucasians	Sweden	1983–2008	Hospital-based	VSCC	FFPE	HMBS PCR analysis	40/130	30.8%	6, 11, 16, 18, 31, 33, 39, 45, 51, 52, 56, 58, 59	PCR
Lee, et al (2016)	Caucasians	United States	1985–2011	Institution based	VSCC	FFPE	β-globin PCR analysis	15/56	27%	16, 18, 27, 33	multiplex PCR
Lindell, et al (2010)	Caucasians	Sweden	2000–2007	Institution based	VSCC	FFPE	Housekeeping gene by PCR	23/75	31%	6, 11, 16, 18, 33	PCR(GP5+/6+ and CPI/IIG)
Menczer, et al (2000)	Caucasians	Israeli	NA	NA	VC	FFPE	NA	9/14	64.2%	16, 18	PCR, HPV negative cases were re-examined with a sensitive primer.
Ngamkham, et al (2016)	Asians	Thailand	2003–2012	Institution based	VC	FFPE	β-globin PCR analysis	16/34	47.1%	6, 11, 16, 18, 31, 33, 35, 45, 58	PCR-EIA
Ordi, et al (2016)	Caucasians	Spain	1980–2011	NA	VSCC	FFPE	NA	184/791	23.3%	NA	SPF10PCR/DEIA/LiPA25 system
Pinto, et al (2004)	Mixed	Brazil	1975–1992	Hospital-based	VC	FFPE	β-globin PCR analysis	38/161	23.6%	6, 11, 16, 18, 45	PCR and DBH (GP5+/GP6+)
Poblet, et al (2010)	Caucasians	Spain	NA	Hospital-based	VC	FFPE	NA	11/37	30.3%	16, 18, 33, 35	PCR(GP5+/GP6+ and My09/My11)
Rakislova, et al (2016)	Caucasians	Spain	NA	NA	VSCC	NA	NA	452/1636	27.6%	NA	NA
Reuschenbach, et al (2013)	Caucasians	Germany	2003–2009	Institution based	VC	FFPE	NA	80/183	43.7%	6, 11, 16, 18, 26, 31, 33, 35, 39, 42, 43, 44, 45, 51, 52, 53, 56, 58, 59, 66, 68, 70, 73, 82	PCR, a multiplex test based on the Luminex technology
Rodrigues, et al (2013)	Mixed	Brazil	1979–2006	Institution-based	VSCC	FFPE	β-globin PCR analysis	34/87	39.1%	16, 18, 31, 33, 35, 42, 45, 53,54, 71, 82, 84	Linear array HPV- test
Rumbold, et al (2012)	Australia	Australia	2007–2009	Institution based	VC	Fresh	β-globin PCR analysis	201/521	38.6%	6, 11, 16, 18	PCR PGMY09/11, Roche Linear Array
Sagdeo, et al (2014)	Caucasians	United States	NA	Institution based	VSCC	FFPE	β-globin PCR analysis	13/17	76.5%	16, 18, 33, 45, 53, 120	PCR (PGMY-GP+-primer system)
Santos, et al (2006)	Caucasians	Spain	1995–2005	Hospital-based	VSCC	FFPE	β-globin PCR analysis	16/92	17.4%	6, 11, 16, 18, 26, 31, 33, 34, 35, 39, 40, 42, 43, 44, 45, 51, 52, 53, 54, 55, 56, 57, 58, 59, 61, 66, 68, 70, 71, 72, 73, 81, 82	PCR(GP5+/GP6+, SPF10)
Serrano, et al (2015)	Mixed	48 countries	NA	Hospital-based	VC	FFPE	NA	489/1709	28.6%	6, 11, 16, 18, 31, 33, 45, 52, 58	SPF-10PCR/DEIA/ LiPA25 System
Siriaunkgul, et al (2014)	Asians	Thailand	2006–2012	Hospital-based	VSCC	FFPE	β-globin PCR analysis	29/47	62%	16, 26, 58, 89	PCR (MY09/11 and GP5+/GP6+.), Linear Array Genotyping Test
Sutton, et al (2008)	Caucasians	United States	1987–2007	Institution-based	VSCC	FFPE	β-globin PCR analysis	81/116	69.8%	6, 16, 18, 26, 33,45, 52, 61	PCR-Linear Array HPV Test
Sznurkowski, et al (2016)	Caucasians	Poland	2002–2007	Institution-based	VC	FFPE	RNAseP gene PCR analysis	38/85	45%	16, 18, 33, 39, 59	SPF10–LiPA25 system
Tsimplaki, et al (2012)	Caucasians	Greece	NA	Hospital-based	VSCC	FFPE	β-globin PCR analysis	3/6	50%	16, 18, 31, 33, 45	PapilloCheck DNA Microarray
Van, et al (2009)	Caucasians	The Netherlands	1988–2005	Institution-based	VSCC	FFPE	β-globin PCR analysis	45/130	34.6%	16, 18, 33, 52, 53,54, 58, 66	PCR and ISH
Wakeham, et al (2017)	Caucasians	UK	2001–2014	Institution-based	VSCC	FFPE	β-globin PCR analysis	32/62	52%	6, 11, 16, 18, 33, 51, 53	Optiplex HPV Genotyping assay

DEIA: DNA enzyme immunoassay; EIA: Enzyme-immunoassay; FFPE: Formalin-fixed paraffin-embedded; ISH: in situ hybridization; IVC: invasive vulvar cancer; IVSCC: invasive squamous cell carcinoma; NA: not available; VC: vulvar cancer; VSCC: vulvar squamous cell carcinoma

Of the 33 studies, 9 studies reported the association between HPV infection and survival outcomes among vulvar cancer patients. The main characteristics of the studies included are summarized in Table 2. The survival indicators mainly included the overall survival (OS, n = 8), progression-free survival (PFS, n = 1), recurrence-free survival (RFS, n = 2), disease-free survival (DFS, n = 2), and disease-specific survival (DSS, n = 2). The HR estimates methods included HR combined with 95% CI and calculated according to the Kaplan-Meier survival curves.

**Table 2:** The main survival indicators of the 9 studies for the meta-analysis.

Study	Survival Indicators	HR type	HR Estimates	Follow-up
Alonso, et al (2011)	OS, DFS	Age-adjusted	HR and 95%CI	3.8 years (range: 0.9 to 5 years)
Kim, et al (2015)	OS, DFS	Age-adjusted	KM	2.8 years (range:0.3 to 18.9 years)
Larsson, et al (2012)	OS	No-adjusted	HR and 95%CI	NA
Lindell, et al (2010)	OS, RFS, DSS	Age and tumor size -adjusted	KM	42.0 months
Pinto, et al (2004)	OS, RFS	Age-adjusted	HR and 95%CI	59.9 months (rang: 1 to 265 months)
Rodrigues, et al (2013)	OS	No-adjusted	KM	5 years
Sznurkowski, et al (2016)	OS	No-adjusted	KM	89.20 months (range: 1.7–189.5 months)
Van, et al (2009)	DSS	No-adjusted	KM	Last: August 1, 2008
Wakeham, et al (2017)	OS, PFS	Age and cancer stage adjusted	HR and 95%CI	5.8years (range 55 days to 14 years)

NA: not available; KM: Kaplan-Meier; OS: Overall survival; PFS: Progression-Free Survival; RFS: Recurrence-Free Survival; DFS: Disease-Free Survival; DSS: Disease-Specific Survival; HR: harzard ratio.

### HPV prevalence in vulvar cancer

**Fig 2:**
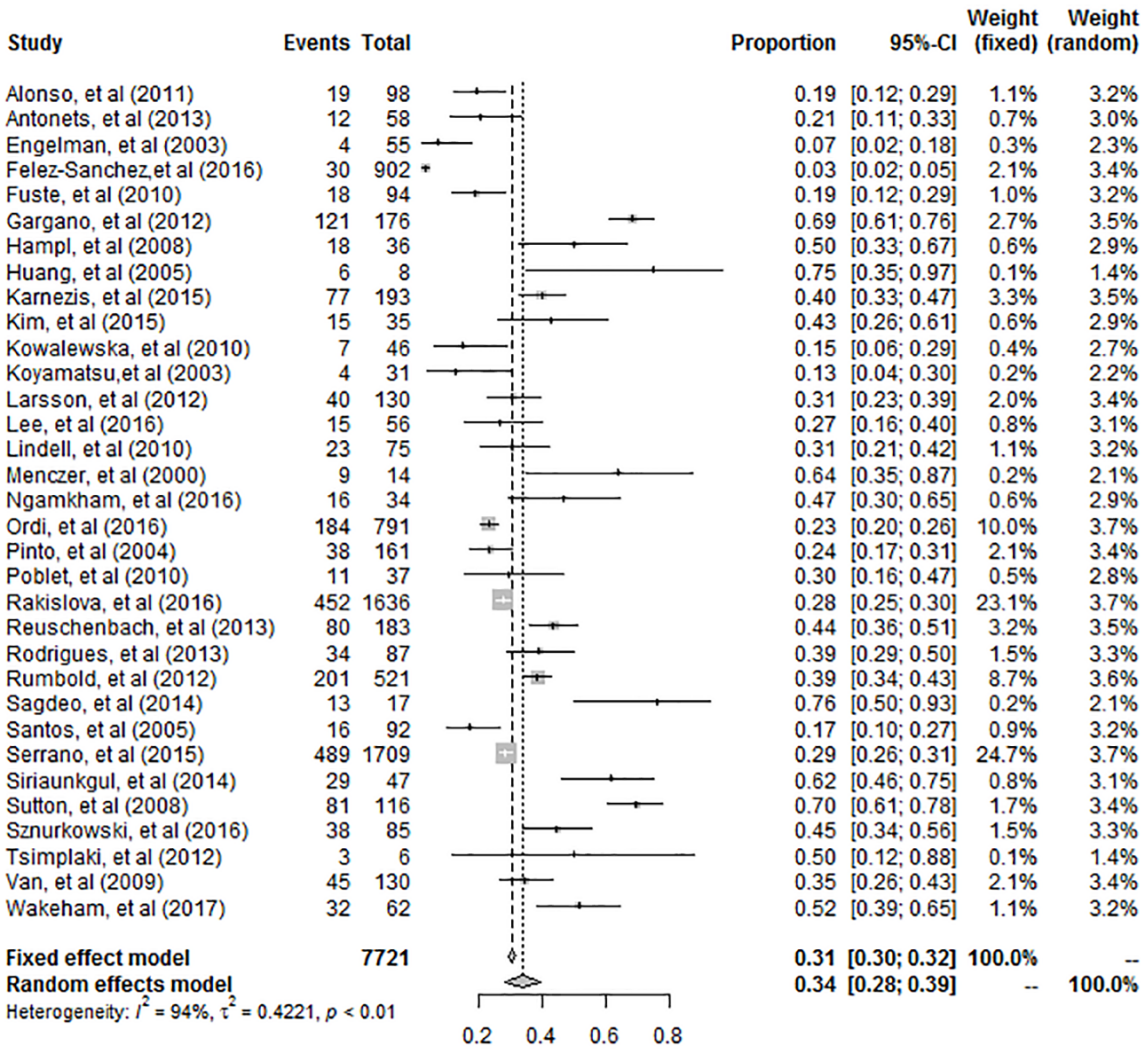
Forest plot of the prevalence of human papillomavirus in vulvar cancer.

A forest plot of the 33 eligible studies is shown in Fig 2. The forest plot showed the pooled prevalence of HPV for all studies with the prevalence of HPV in vulvar cancer for each study. The prevalence of HPV in vulvar cancer varied from 3.3% to as high as 76.5%. From the results of the meta-analysis, the pooled prevalence of HPV in vulvar cancer was 33.7% (95% CI: 28.5%-39.4%).

**Fig 3:**
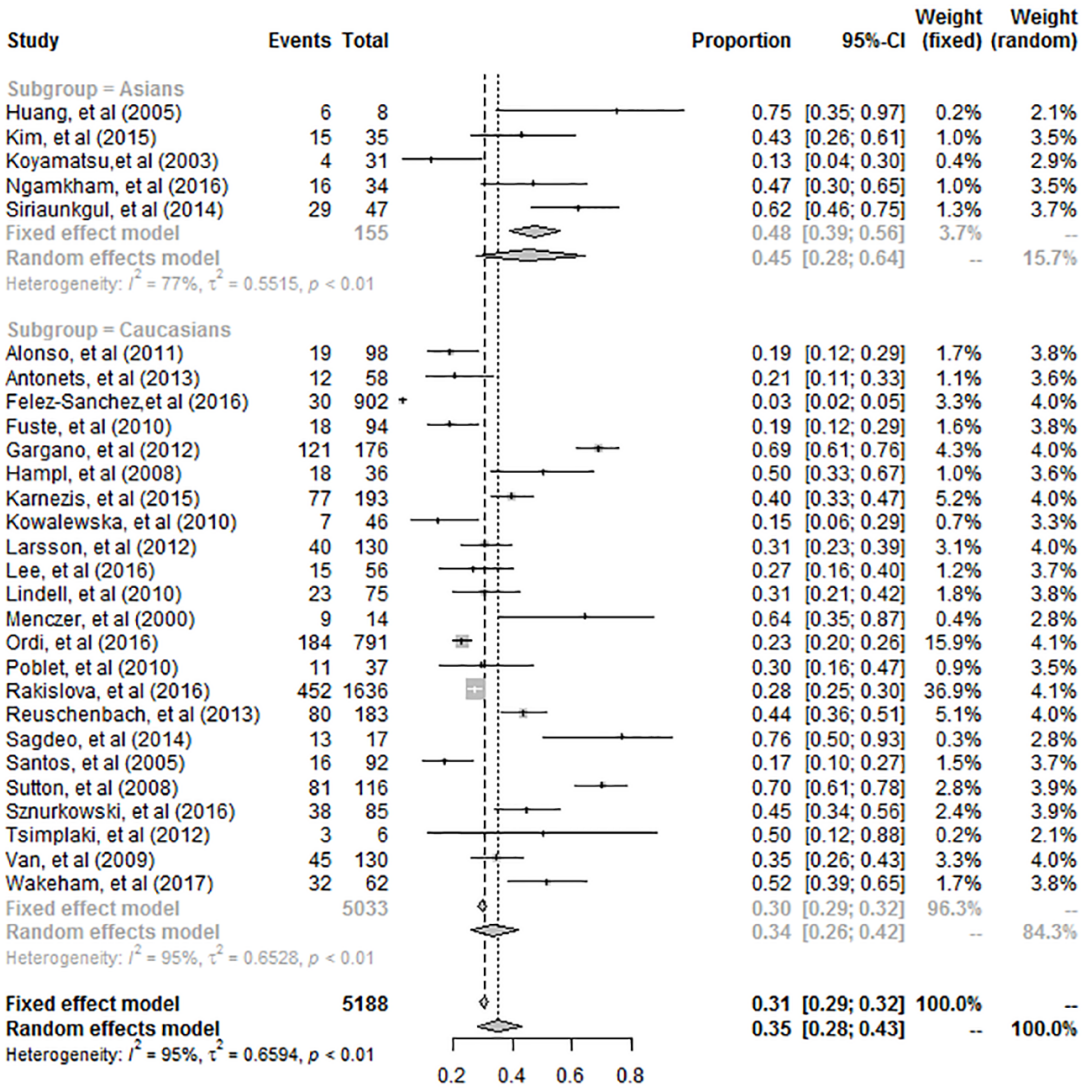
Forest plot for the subgroup analysis of ethnicity.

The subgroup analysis was performed depending on the ethnicity (Fig 3). The pooled prevalence of HPV in Asian populations was 45% (95% CI: 28%-64%), whereas it was 34% (95% CI: 26%–42%) in Caucasian populations. There was no significant difference in HPV prevalence between the two populations (*P* = 0.2582).

### HPV status and survival outcomes in vulvar cancer

**Fig 4:**
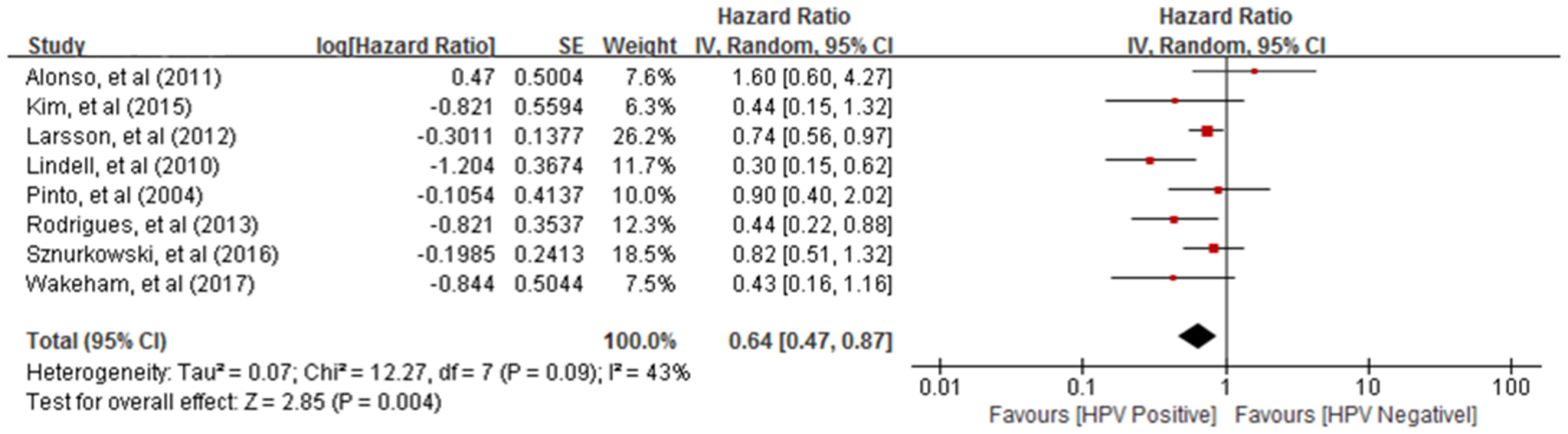
Forest plot for the association between human papillomavirus and overall survival in patients with vulvar cancer.

**Fig 5:**
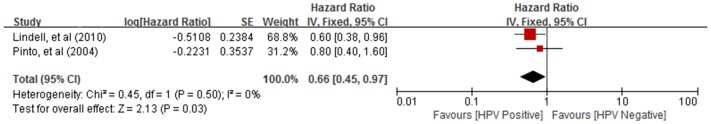
Forest plot for the association between human papillomavirus and recurrence-free survival in patients with vulvar cancer.

**Fig 6:**
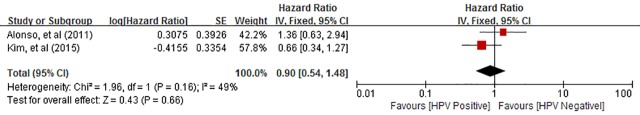
Forest plot for the association between human papillomavirus and disease-free survival in patients with vulvar cancer.

**Fig 7:**
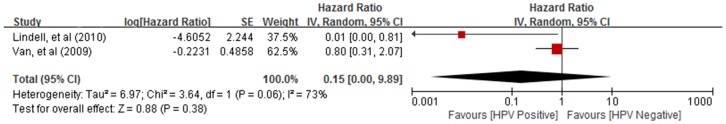
Forest plot for the association between human papillomavirus and disease-specific survival in patients with vulvar cancer.

Nine studies provided data on the association between HPV status and survival outcomes in vulvar cancer patients. The pooled analysis showed that the HPV-positive vulvar cancer was associated with better OS (HR = 0.64, 95% CI: 0.47–0.87; *P* = 0.004; Fig 4) and RFS (HR = 0.66, 95% CI: 0.45–0.97; *P* = 0.03; Fig 5) compared with their HPV-negative counterparts. In addition, HPV-positivity tended to be associated with better DFS (HR = 0.90, 95% CI: 0.54–1.48; *P* = 0.66; Fig 6), and DSS (HR = 0.15, 95% CI: 0.00–9.89; *P* = 0.38; Fig 7).

### Correlation between HPV prevalence and the OS in vulvar cancer

**Fig 8:**
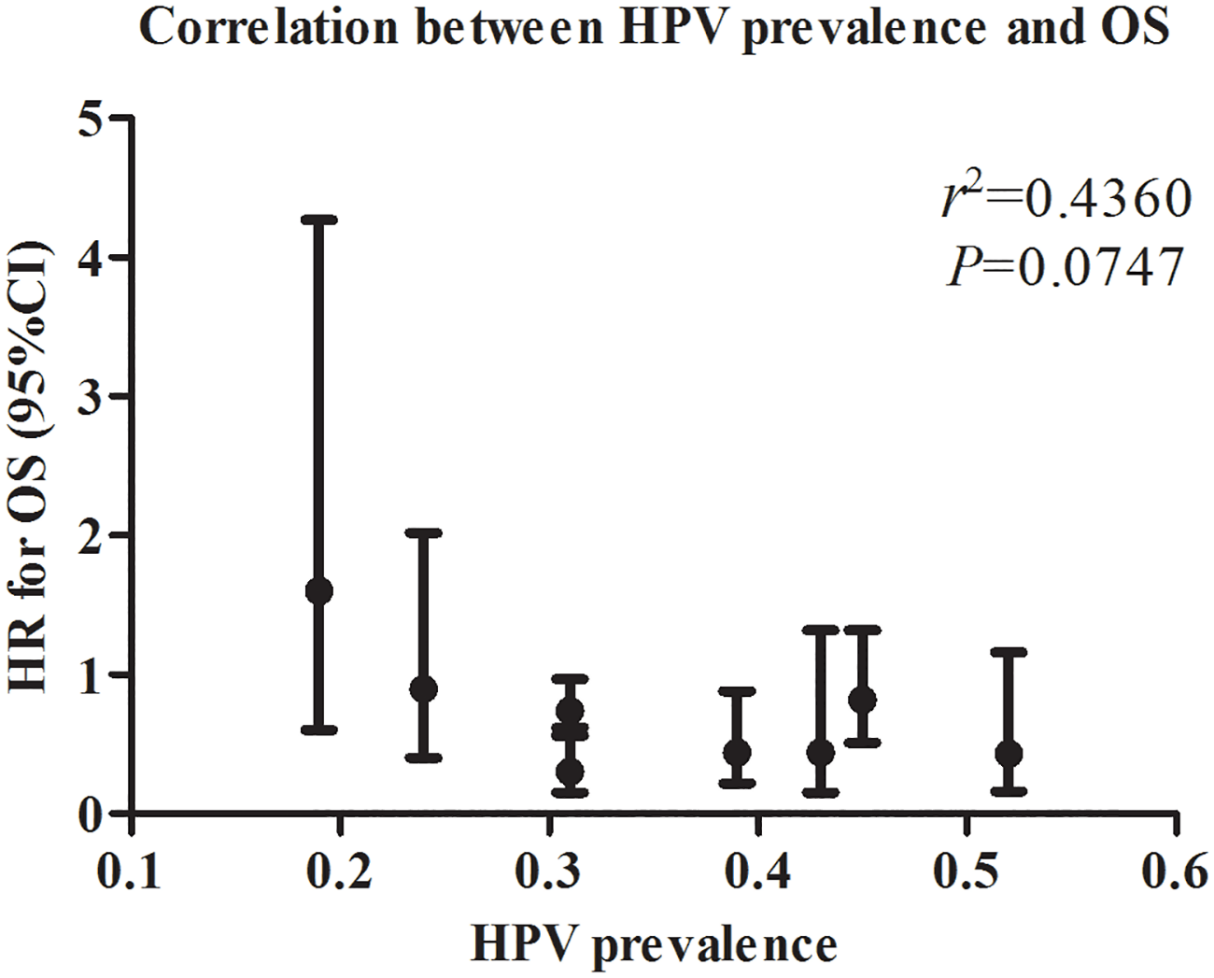
Correlation between HPV prevalence and the OS in vulvar cancer for the 8 studies.

To investigate the correlation between HPV prevalence and the OS in vulvar cancer for the 8 studies, we had plotted the prevalence and the OS (with 95%CI bars) for the 8 studies in a 2 dimension graph (Fig 8). The results (*r*^2^ = 0.4360, *P* = 0.0747) showed that there wasn`t a significant correlation between the prevalence and the OS in vulvar cancer for the 8 studies in our meta-analysis.

### Qualitative assessment

Quality assessment of the 33 eligible studies is shown in S2 Table. The average NOS score of the eligible studies was 7.03 (ranged from 6 to 8), which indicated that the majority of the eligible studies were high quality.

### Sensitivity analysis

A sensitivity analysis was performed for assessing the results of this meta-analysis (data not shown). Results of the sensitivity analysis showed that no significant alteration of the pooled incidence and HRs existed after removing a single study one by one, which indicated that the results of the prevalence and prognostic value of HPV in vulvar cancer were relatively stable and reliable.

### Publication bias

**Fig 9:**
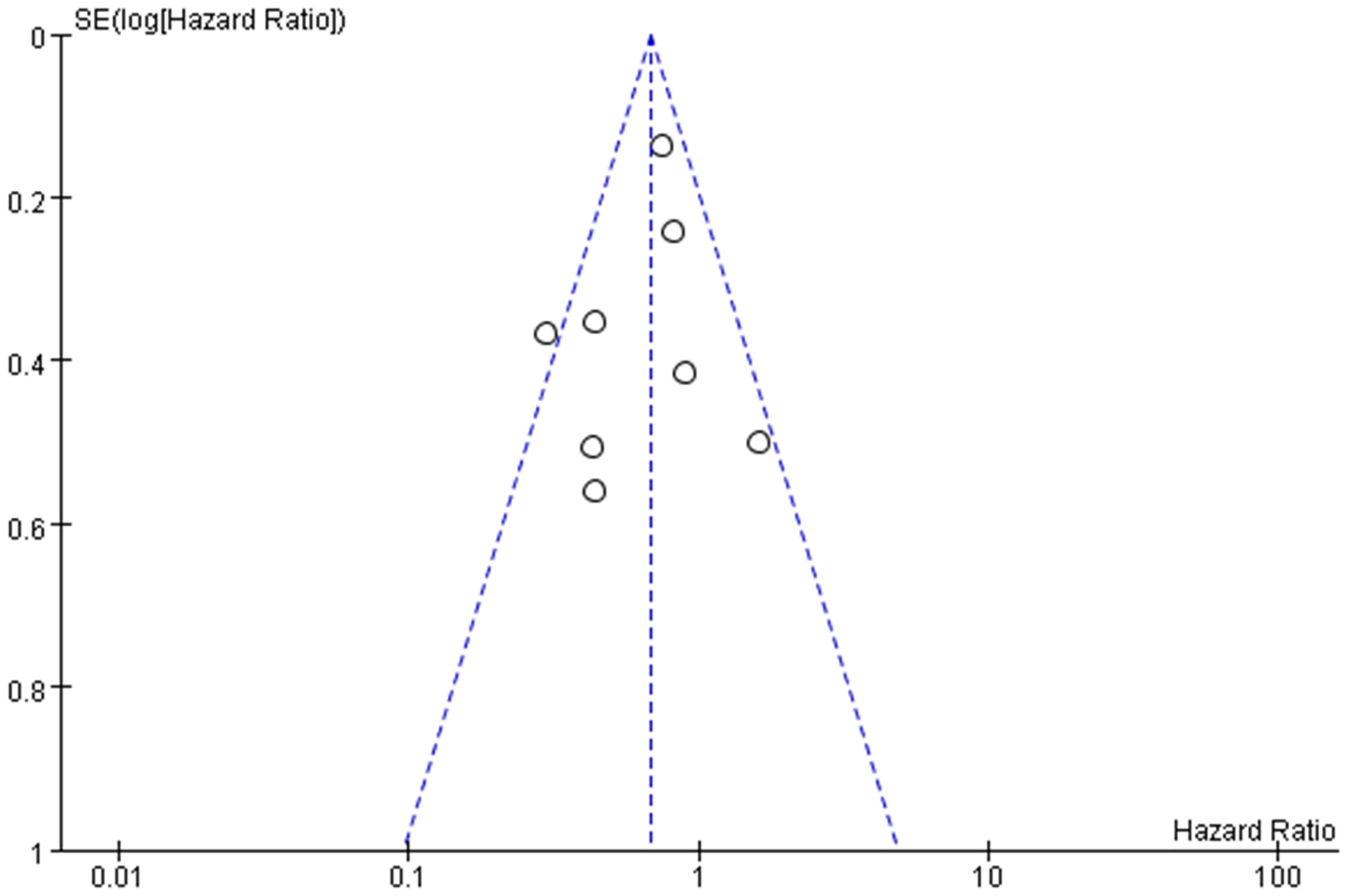
Funnel plot of prognostic value of human papillomavirus in vulvar cancer for publication bias.

The publication bias in the eligible studies, reporting survival outcomes of vulvar cancer was assessed by the funnel plot and Egger’s test. The shape of the funnel plot was approximately symmetrical (Fig 9). Additionally, the Egger’s test suggested that no publication bias was existed (*P* = 0.487).

## Discussion

Vulvar cancer is a rare type of gynecological cancer among women worldwide and is more commonly diagnosed in older women (over 65 years) (5–7, 23). Based on the discovery and identification of prognostic biomarkers, the therapeutic approaches of vulvar cancer will evolve from standard radical resections to more individualized approaches (35, 36). Because the definition of new prognostic variables might result in further individualization of the treatment of vulvar cancer, it would be useful to confirm more prognostic biomarkers of vulvar cancer to guide the individualized treatment.

HPV was found to have a causal role in some types of gynecological cancers such as cervical cancer [51]. It was reported that persistent high-risk HPV infection was the essential cause for the development of vulvar abnormal lesions or progression of vulvar cancer [52]. Furthermore, several studies found the prognostic significance of HPV infection for vulvar cancer; however, with controversial results. Meanwhile, the prevalence rates of HPV were also inconsistent in the reported studies. Although a previous systematic review and meta-analysis had estimated the pooled prevalence of HPV in vulvar cancer, this study ignored the prognostic value of HPV in vulvar cancer (53). Thus, we conducted a systematic review and meta-analysis to investigate the prevalence rates of HPV and to clarify the real association between the HPV status and survival outcomes in patients with vulvar cancer.

In this meta-analysis containing 33 studies and 7,721 cases of vulvar cancer, the pooled prevalence rate of HPV was 34% (95% CI: 28%–39%) with large study heterogeneity (*I*^*2*^ = 94%, *P*<0.01). The greater diverging prevalence rates of HPV positivity in these studies could generally be explained by different HPV detection methods (PCR, ISH, etc.), different case selection, or focusing on different types of HPV. In contrast to the established role of HPV as a risk factor, a significantly better survival outcome for women with HPV-positive tumors was found, compared to women with HPV-negative tumors. The pooled HRs of the associations between HPV status and OS, RFS indicated risk factors of 0.64 and 0.66 with significant *P* values, respectively. In addition, HPV-positive status tended to be associated with better DFS and DSS, although there was no statistical difference. This lack of significance could be partially explained by the small number of articles, as only two studies were included.

The drivers as to why HPV-associated vulvar cancer is associated with improved outcomes compared to their HPV negative counterparts are not fully established. Some factors, such as age at diagnosis, tumor size, subtype and clinical stage, morphology and histopathologic grade are important to predict tumor prognosis as well. However, we could not analyze the impact of each factor on the prognostic value of HPV in vulvar cancer patients because of the limitation of data. We could not determine whether the HPV status is an independent prognostic factor for vulvar cancer. Some studies found that patients with HPV-positive tumors had better survival, when adjusted for age and tumor size, than patients with HPV-negative tumors [32]. However, it was also reported that HPV-positive cases showed better OS than those of HPV-negative ones, while multivariate analysis did not show an independent prognostic significance [40, 50]. As the HRs in the majority of the eligible studies were age-adjusted, our results might be with a relative higher credibility indicating HPV-positive vulvar cancer women might relatively have a better survival than HPV-negative ones. However, it needs more evidence to support this conclusion.

Vulvar cancer develops through 2 distinct molecular pathways, one involving high-risk HPV infection and often observed in the younger patients less than 50 years old, and the other through early p53 suppressor gene mutation and often observed in the elderly patients. We speculate that the different pathogenesis and characteristics of the two types of vulvar cancer may partially explain the different survival outcomes. Notably HPV-positive (and p16-positive) patients were reported to be less likely to have recurrence and there were no vulvar cancer related deaths, whereas p53-mutant positive patients had a greater probability of recurrence and were significantly more likely to die from vulvar cancer [53]. Moreover, HPV-positivity was more common in younger patients, while HPV-negativity was more common in elder patients of vulvar cancer. Age was proved to be an effective prognostic indicator in vulvar cancer [10, 27]. Furthermore, other factors associated with HPV status could also influence the prognosis of vulvar cancer patients. For example, a recent study conducted by Rodrigues, et al. indicated that loss of β-catenin and high Slug, Snail and Twist expression was associated with HPV-negative tumors [40]. The alterations in β-catenin and Slug expression may increase the risk of deeper invasion and metastasis characteristic due to a potentially more aggressive behavior of the tumor cells in the tumor front. Because of the lack of EMT-like events, the patients with HPV-positive tumors may usually have better prognosis. Meanwhile, HPV-negative tumors, which develop EMT-like events, would increase capability of invasion and progression, therefore leading to worse prognosis and poorer outcomes.

There are some limitations of this meta-analysis. Firstly, some relevant studies were excluded in the meta-analysis due to incomplete raw data or publication limitations. Secondly, studies in other databases might have been lost. Thirdly, among the studies that reported the association between the HPV infection and survival outcomes in vulvar cancer, several studies that needed to calculate HR and its related 95% CI might have caused potential bias and imprecise values. Additionally, not all of the primary studies included in our meta-analysis analyzed the other many important prognostic factors comprehensively. Therefore, we could not determine whether HPV status is an independent prognostic factor for vulvar cancer. Furthermore, due to the large numbers of variables such as HPV types, detection methods and so on in studies about HPV prevalence and the limited studies about the prognostic value of HPV on vulvar cancer, we couldn`t determine the underlying causes of heterogeneity in prevalence and prognostic value.

In conclusion, our study is the first meta-analysis to explore the prognostic value of HPV infection in vulvar cancer. We demonstrated a high prevalence of HPV-positivity in vulvar cancer cases which was similar with the previous study (53). The HPV status may act as a biomarker for predicting the prognosis of patients with vulvar cancer. More large-scale and well-designed studies are needed to confirm whether HPV status is an independent prognostic factor for vulvar cancer in the future.

## Supporting information

S1 Table The search strategy for the prevalence and prognostic value of human papillomavirus in vulvar cancer. (DOC)

S2 Table Application of the quality assessment tool NOS to the studies included in the meta-analysis. (XLSX)

## References

[1] Siegel RL, Miller KD, Jemal A. Cancer Statistics, 2017. CA Cancer J Clin. 2017;67(1):7–30. 10.3322/caac.21387 28055103

[2] Jones RW, Baranyai J, Stables S. Trends in squamous cell carcinoma of the vulva: the influence of vulvar intraepithelial neoplasia. Obstet Gynecol. 1997;90(3):448–452. 9277660 10.1016/s0029-7844(97)00298-6

[3] Baandrup L, Varbo A, Munk C, Johansen C, Frisch M, Kjaer SK. In situ and invasive squamous cell carcinoma of the vulva in Denmark 1978-2007-a nationwide population-based study. Gynecol Oncol. 2011;122(1):45–49. 10.1016/j.ygyno.2011.03.016 21474166

[4] Judson PL, Habermann EB, Baxter NN, Durham SB, Virnig BA. Trends in the incidence of invasive and in situ vulvar carcinoma. Obstet Gynecol. 2006;107(5):1018 10.1097/01.AOG.0000210268.57527.a1 16648405

[5] Joura EA, Losch A, Haider-Angeler MG, Breitenecker G, Leodolter S. Trends in vulvar neoplasia. Increasing incidence of vulvar intraepithelial neoplasia and squamous cell carcinoma of the vulva in young women. J Reprod Med. 2000;45(8):613–615. 10986677

[6] Chhabra S, Bhavani M, Deshpande A. Trends of vulvar cancer. J Obstet Gynaecol. 2014;34(2):165 10.3109/01443615.2013.834310 24456439

[7] Stroup AM, Harlan LC, Trimble EL. Demographic, clinical, and treatment trends among women diagnosed with vulvar cancer in the United States. Gynecol Oncol. 2008;108(3):577–583. 10.1016/j.ygyno.2007.11.011 18155274 PMC2350205

[8] Del Pino M, Rodriguez-Carunchio L, Ordi J. Pathways of vulvar intraepithelial neoplasia and squamous cell carcinoma. Histopathology. 2013;62(1):161–175. 10.1111/his.12034 23190170

[9] Koh WJ, Greer BE, Abu-Rustum NR, Campos SM, Cho KR, Chon HS, et al Vulvar Cancer, Version 1.2017, NCCN Clinical Practice Guidelines in Oncology. J Natl Compr Canc Netw. 2017;15(1):92–120. 28040721 10.6004/jnccn.2017.0008

[10] Zweizig S, Korets S, Cain JM. Key concepts in management of vulvar cancer. Best Pract Res Clin Obstet Gynaecol. 2014;28(7):959–966. 10.1016/j.bpobgyn.2014.07.001 25151473

[11] de Sanjose S, Alemany L, Ordi J, Tous S, Alejo M, Bigby SM, et al Worldwide human papillomavirus genotype attribution in over 2000 cases of intraepithelial and invasive lesions of the vulva. Eur J Cancer. 2013;49(16):3450–3461. 10.1016/j.ejca.2013.06.033 23886586

[12] Munoz N, Castellsague X, de Gonzalez AB, Gissmann L. Chapter 1: HPV in the etiology of human cancer. Vaccine. 2006;24 Suppl 3:S3/1–10. 10.1016/j.vaccine.2006.05.115 16949995

[13] de Villiers EM, Fauquet C, Broker TR, Bernard HU, zur Hausen H. Classification of papillomaviruses. Virology. 2004;324(1):17–27. 10.1016/j.virol.2004.03.033 15183049

[14] Munoz N, Bosch FX, de Sanjose S, Herrero R, Castellsague X, Shah KV, et al Epidemiologic classification of human papillomavirus types associated with cervical cancer. N Engl J Med. 2003;348(6):518–527. 10.1056/NEJMoa021641 12571259

[15] Stang A. Critical evaluation of the Newcastle-Ottawa scale for the assessment of the quality of nonrandomized studies in meta-analyses. Eur J Epidemiol. 2010;25(9):603–605. 10.1007/s10654-010-9491-z 20652370

[16] Parmar MK, Torri V, Stewart L. Extracting summary statistics to perform meta-analyses of the published literature for survival endpoints. Stat Med. 1998;17(24):2815–2834. 9921604 10.1002/(sici)1097-0258(19981230)17:24<2815::aid-sim110>3.0.co;2-8

[17] Tierney JF, Stewart LA, Ghersi D, Burdett S, Sydes MR. Practical methods for incorporating summary time-to-event data into meta-analysis. Trials. 2007;8:16 10.1186/1745-6215-8-16 17555582 PMC1920534

[18] Alonso I, Fuste V, del Pino M, Castillo P, Torne A, Fuste P, et al Does human papillomavirus infection imply a different prognosis in vulvar squamous cell carcinoma? Gynecol Oncol. 2011;122(3):509–514. 10.1016/j.ygyno.2011.05.016 21652058

[19] Antonets AV, Nerodo G, Dvadnenko KV, Zykova TA, Ohremenko NV, Nerodo EA. Detection of human papillomavirus in vulva cancer tissue. J Clin Oncol. 2013;31(15):1904–1911.23589547

[20] Engelman DE, Andrade LA, Vassallo J. Human papillomavirus infection and p53 protein expression in vulvar intraepithelial neoplasia and invasive squamous cell carcinoma. Braz J Med Biol Res. 2003;36(9):1159–1165. 12937780 10.1590/s0100-879x2003000900003

[21] Felez-Sanchez M, Vergara M, de Sanjose S, Castellsague X, Alemany L, Bravo IG. Searching beyond the usual papillomavirus suspects in squamous carcinomas of the vulva, penis and head and neck. Infect Genet Evol. 2016;45:198–204. 10.1016/j.meegid.2016.09.003 27600594

[22] Fuste V, Alonso I, Castillo P, al e. Squamous cell carcinoma of the vulva: Clinicopathological correlations and prognostic significance of HPV infection and FIGO staging. Lab Invest. 2010;90(S1):243A.

[23] Gargano JW, Wilkinson EJ, Unger ER, Steinau M, Watson M, Huang Y, et al Prevalence of human papillomavirus types in invasive vulvar cancers and vulvar intraepithelial neoplasia 3 in the United States before vaccine introduction. J Low Genit Tract Dis. 2012;16(4):471–479. 10.1097/LGT.0b013e3182472947 22652576 PMC5553114

[24] Hampl M, Deckers-Figiel S, Hampl JA, Rein D, Bender HG. New aspects of vulvar cancer: changes in localization and age of onset. Gynecol Oncol. 2008;109(3):340–345. 10.1016/j.ygyno.2008.01.041 18407339

[25] Huang FY, Kwok YK, Lau ET, Tang MH, Ng TY, Ngan HY. Genetic abnormalities and HPV status in cervical and vulvar squamous cell carcinomas. Cancer Genet Cytogenet. 2005;157(1):42–48. 10.1016/j.cancergencyto.2004.06.002 15676146

[26] Karnezis A, Cheng A, Mcalpine J, al. e. Assessing the accuracy of histomorphology in distinguishing between HPV-positive and HPV-negative vulvar squamous cell carcinomas. Mod Pathol. 2015;28(S2):293A.

[27] Kim Y, Kim JY, Kim JY, Lee NK, Kim JH, Kim YB, et al Treatment outcomes of curative radiotherapy in patients with vulvar cancer: results of the retrospective KROG 1203 study. Radiat Oncol J. 2015;33(3):198–206. 10.3857/roj.2015.33.3.198 26484303 PMC4607573

[28] Kowalewska M, Szkoda MT, Radziszewski J, Ptaszynski K, Bidzinski M, Siedlecki JA. The frequency of human papillomavirus infection in polish patients with vulvar squamous cell carcinoma. Int J Gynecol Cancer. 2010;20(3):434–437. 10.1111/IGC.0b013e3181d320f1 20375810

[29] Koyamatsu Y, Yokoyama M, Nakao Y, Fukuda K, Saito T, Matsukuma K, et al A comparative analysis of human papillomavirus types 16 and 18 and expression of p53 gene and Ki-67 in cervical, vaginal, and vulvar carcinomas. Gynecol Oncol. 2003;90(3):547–551. 13678722 10.1016/s0090-8258(03)00401-3

[30] Larsson GL, Helenius G, Andersson S, Elgh F, Sorbe B, Karlsson MG. Human papillomavirus (HPV) and HPV 16-variant distribution in vulvar squamous cell carcinoma in Sweden. Int J Gynecol Cancer. 2012;22(8):1413–1419. 10.1097/IGC.0b013e31826a0471 23013732

[31] Lee LJ, Howitt B, Catalano P, Tanaka C, Murphy R, Cimbak N, et al Prognostic importance of human papillomavirus (HPV) and p16 positivity in squamous cell carcinoma of the vulva treated with radiotherapy. Gynecol Oncol. 2016;142(2):293–298. 10.1016/j.ygyno.2016.05.019 27210818

[32] Lindell G, Nasman A, Jonsson C, Ehrsson RJ, Jacobsson H, Danielsson KG, et al Presence of human papillomavirus (HPV) in vulvar squamous cell carcinoma (VSCC) and sentinel node. Gynecol Oncol. 2010;117(2):312–316. 10.1016/j.ygyno.2009.12.031 20138657

[33] Menczer J, Fintsi Y, Arbel-Alon S, Tell L, Friedman E, Jackman A, et al The presence of HPV 16, 18 and p53 immunohistochemical staining in tumor tissue of Israeli Jewish women with cervical and vulvar neoplasia. Eur J Gynaecol Oncol. 2000;21(1):30–34. 10726615

[34] Ngamkham J, Boonmark K, Phansri T. Detection and Type-Distribution of Human Papillomavirus in Vulva and Vaginal Abnormal Cytology Lesions and Cancer Tissues from Thai Women. Asian Pac J Cancer Prev. 2016;17(3):1129–1134. 27039737 10.7314/apjcp.2016.17.3.1129

[35] Ordi J, Rakislova NC, OOC., al. e. Histological characteristics of HPV-associated and-independent squamous cell carcinomas of the vulva: A study of 791 cases. Mod Pathol. 2016;29(S2):300A. 10.1002/ijc.31006 28815579

[36] Pinto AP, Schlecht NF, Pintos J, Kaiano J, Franco EL, Crum CP, et al Prognostic significance of lymph node variables and human papillomavirus DNA in invasive vulvar carcinoma. Gynecol Oncol. 2004;92(3):856–865. 10.1016/j.ygyno.2003.11.052 14984953

[37] Poblet E, Pascual-Martín A, Pariente-Martín M, Chiarri-Rodrigo J, Vera-Berón R, Godínez JM, editors. Prevalence and Genotype Identification of HPV Infection in Penile vs. Vulvar Carcinomas. Meeting of the United-States-And-Canadian-Academy-Of-Pathology; 2010.

[38] Rakislova N, Clavero O, Alemany L, Saco A, Quirós B, Lloveras B, et al "Histological characteristics of HPV-associated and -independent squamous cell carcinomas of the vulva: A study of 1594 cases". Int J Cancer. 2017;141. 10.1002/ijc.31006 28815579

[39] Reuschenbach M, Roos J, Panayotopoulos D, Baldus SE, Schnurch HG, Berger A, et al Characterization of squamous cell cancers of the vulvar anterior fourchette by human papillomavirus, p16INK4a, and p53. J Low Genit Tract Dis. 2013;17(3):289–297. 10.1097/LGT.0b013e31826f2b2b 23645067

[40] Rodrigues IS, Lavorato-Rocha AM, de MMB, Stiepcich MM, de Carvalho FM, Baiocchi G, et al Epithelial-mesenchymal transition-like events in vulvar cancer and its relation with HPV. Br J Cancer. 2013;109(1):184–194. 10.1038/bjc.2013.273 23778524 PMC3721089

[41] Rumbold AR, Tan SE, Condon JR, Taylor-Thomson D, Nickels M, Tabrizi SN, et al Investigating a cluster of vulvar cancer in young women: a cross-sectional study of genital human papillomavirus prevalence. BMC Infect Dis. 2012;12:243 10.1186/1471-2334-12-243 23040203 PMC3507832

[42] Sagdeo A, Gormley RH, Abuabara K, Tyring SK, Rady P, Elder DE, et al The diagnostic challenge of vulvar squamous cell carcinoma: Clinical manifestations and unusual human papillomavirus types. J Am Acad Dermatol. 2014;70(3):586–588. 10.1016/j.jaad.2013.11.027 24528909 PMC4349358

[43] Santos M, Landolfi S, Olivella A, Lloveras B, Klaustermeier J, Suarez H, et al p16 overexpression identifies HPV-positive vulvar squamous cell carcinomas. Am J Surg Pathol. 2006;30(11):1347–1356. 10.1097/01.pas.0000213251.82940.bf 17063073

[44] Serrano B, de Sanjose S, Tous S, Quiros B, Munoz N, Bosch X, et al Human papillomavirus genotype attribution for HPVs 6, 11, 16, 18, 31, 33, 45, 52 and 58 in female anogenital lesions. Eur J Cancer. 2015;51(13):1732–1741. 10.1016/j.ejca.2015.06.001 26121913

[45] Siriaunkgul S, Settakorn J, Sukpan K, Srisomboon J, Utaipat U, Lekawanvijit S, et al HPV detection and genotyping in vulvar squamous cell carcinoma in northern Thailand. Asian Pac J Cancer Prev. 2014;15(8):3773–3778. 24870792 10.7314/apjcp.2014.15.8.3773

[46] Sutton BC, Allen RA, Moore WE, Dunn ST. Distribution of human papillomavirus genotypes in invasive squamous carcinoma of the vulva. Mod Pathol. 2008;21(3):345–354. 10.1038/modpathol.3801010 18192967

[47] Sznurkowski JJ, Anton Ż, Wojciech B. The overexpression of p16 is not a surrogate marker for high-risk human papilloma virus genotypes and predicts clinical outcomes for vulvar cancer. Bmc Cancer. 2016;16(1):465. 27411473 10.1186/s12885-016-2503-y PMC4944532

[48] Tsimplaki E, Argyri E, Michala L, Kouvousi M, Apostolaki A, Magiakos G, et al Human papillomavirus genotyping and e6/e7 mRNA expression in greek women with intraepithelial neoplasia and squamous cell carcinoma of the vagina and vulva. J Oncol. 2012;2012:893275 10.1155/2012/893275 22187556 PMC3236520

[49] van de Nieuwenhof HP, van Kempen LC, de Hullu JA, Bekkers RL, Bulten J, Melchers WJ, et al The etiologic role of HPV in vulvar squamous cell carcinoma fine tuned. Cancer Epidemiol Biomarkers Prev. 2009;18(7):2061–2067. 10.1158/1055-9965.EPI-09-0209 19567503

[50] Wakeham K, Kavanagh K, Cuschieri K, Millan D, Pollock KG, Bell S, et al HPV status and favourable outcome in vulvar squamous cancer. Int J Cancer. 2017;140(5):1134–1146. 10.1002/ijc.30523 27864932

[51] Goodman A. HPV testing as a screen for cervical cancer. BMJ. 2015;350:h2372 10.1136/bmj.h2372 26126623

[52] Guo YL, Geng L, You K, Qiao J, Liu CR. [Preliminary study of vulvar and vaginal intraepithelial neoplasias]. Beijing Da Xue Xue Bao Yi Xue Ban. 2009;41(5):561–564. 19829675

[53] Hay CM, Lachance JA, Lucas FL, Smith KA, Jones MA. Biomarkers p16, Human Papillomavirus and p53 Predict Recurrence and Survival in Early Stage Squamous Cell Carcinoma of the Vulva. J Low Genit Tract Dis. 2016;20(3):252–256. 10.1097/LGT.0000000000000182 26855143

