# Supplementary material for: Prevalence of human papillomavirus and its prognostic value in vulvar cancer: A systematic review and meta-analysis
Source: PLoS One. 2018 Sep 26;13(9):e0204162. doi: 10.1371/journal.pone.0204162 (PMC6157864; doi:10.1371/journal.pone.0204162)
Supplement: S1 Table — (DOC) [file pone.0204162.s001.doc]

S1 Table. The search strategy for the prevalence and prognostic value of human papillomavirus in vulvar cancer

| **Ovid EMBASE**   1. (malignan$ OR neoplasm$ OR cancer$ OR carcinoma$ OR adenocarcinoma$ OR tumour$ OR tumor$ OR malign$).tw 2. (vulva OR vulvar OR vulvectomy OR genito urinary OR genito-urinary OR genitourinary OR reproductive OR pelvic OR genital$ OR genitalia OR inguinal OR groin OR perineum OR perineal OR squamous cell carcinoma of the vulva).tw 3. (human papilloma virus) OR (human papilloma viruses) OR (human papillomavirus) OR (human papillomaviruses) OR (human papillomavir$) OR (human papillomaviridae) OR (human papillomavirus infection$) OR HPV 4. animal OR animals OR cells OR *in vitro* 5. review 6. 1 and 2 and 3 not 4 not 5 |
| --- |
| **Pubmed**  1. carcinoma OR carcinom* OR cancer OR cancer* OR malignancy OR malignancies OR malign* OR adenocarcinoma OR adenocarcinom* OR neoplasm OR neoplasm* OR neoplasms OR carcinoma  2. vulva OR vulvar OR vulvectomy OR (genito urinary) OR genito-urinary OR genitourinary OR reproductive OR pelvic OR genital OR genitals OR genitalia OR inguinal OR groin OR perineum OR perineal OR (squamous cell carcinoma of the vulva)  3. (papilloma virus) OR (papilloma viruses) OR papillomavirus OR papillomaviruses OR papillomavir* OR papillomaviridae OR papillomavirus infections OR HPV  4. human OR humans  5. animal* OR cells OR *in vitro*  6. review  7. 1 and 2 and 3 and 4 not 5 not 6 |
| **Web of Science**  1.(carcinoma OR carcinom* OR cancer OR cancer* OR malignancy OR malignancies OR malign* OR adenocarcinoma OR adenocarcinom* OR neoplasm OR neoplasm* OR neoplasms OR carcinoma)  2. vulva OR vulvar OR vulvectomy OR “genito urinary” OR genito-urinary OR genitourinary OR reproductive OR pelvic OR genital OR genitals OR genitalia OR inguinal OR groin OR perineum OR perineal OR “squamous cell carcinoma of the vulva”  3. papilloma virus OR papilloma viruses OR papillomavirus OR papillomaviruses OR papillomavir* OR papillomaviridae OR papillomavirus infections OR HPV  4. human OR humans  5. animal* OR cells OR *in vitro*  6. review  7. 1 and 2 and 3 and 4 not 5 not 6 |
| **Cochrane library**  #1 carcinoma OR carcinom* OR cancer OR cancer* OR malignancy OR malignancies OR malign* OR adenocarcinoma OR adenocarcinom* OR neoplasm OR neoplasm* OR neoplasms OR carcinoma  #2 vulva OR vulvar OR vulvectomy OR “genito urinary” OR genito-urinary OR genitourinary OR reproductive OR pelvic OR genital OR genitals OR genitalia OR inguinal OR groin OR perineum OR perineal OR “squamous cell carcinoma of the vulva”  #3 papilloma virus OR papilloma viruses OR papillomavirus OR papillomaviruses OR papillomavir* OR papillomaviridae OR papillomavirus infections OR HPV  #4 human OR humans  #5 animal* OR cells OR *in vitro*  *#*6 review  #7 (#1 and #2 and #3 and #4 not #5 not #6) |
